# Gout of feet and ankles in different stages: The potentiality of a new semiquantitative DECT scoring system in monitoring urate deposition

**DOI:** 10.1097/MD.0000000000032722

**Published:** 2023-01-20

**Authors:** Huanhuan Zhong, Minghua Wang, Heng Zhang, Zhitian Huang, Baochang Zou, Guannan Zou, Nanai Xie, Yiwen Liang, Yuhui Zhu, Wanling Ma

**Affiliations:** a Department of Radiology, The Second Affiliated Hospital, School of Medicine, The Chinese University of Hong Kong, Shenzhen, Guangdong Province, China; b Department of Pathology, The Second Affiliated Hospital, School of Medicine, The Chinese University of Hong Kong, Shenzhen, Guangdong Province, China.

**Keywords:** DECT scoring system, disease duration, dual-energy computed tomography (DECT), gout, urate crystals

## Abstract

The purpose of this study was to investigate the diagnostic performance of a novel semi-quantitative dual-energy computed tomography (DECT) scoring system in monitoring urate deposition. This study included 287 patients with gout. All patients underwent ankle/foot DECT scans. DECT scores at different stages were compared and their diagnostic efficacies evaluated. Associations between DECT scores and clinical variables were evaluated. Gouts with positive DECT results in early, middle, and late stages were 78.5 %, 81.4 %, and 95.8 %, respectively (all *P *> .05). The total and ankle/midfoot DECT scores at different stages significantly increased with disease duration (all *P *< .05). DECT scores of 4 regions excluding the first metatarsophalangeal joint in early and middle stages were lower than those in late stage (all *P *< .05). DECT scores achieved excellent diagnostic performance for differentiating gout in early stage from middle and late stages (area under the curve, 0.923 and 0.949), with high sensitivity, specificity, positive predictive value, and negative predictive value (all > 85 %). Total DECT score was highly positively correlated with the volume of urate crystals (*R* = 0.873, *P *< .001). Disease duration, serum uric acid level, bone erosion, and Achilles tendon involvement significantly affected total DECT scores (all *P *< .01). In conclusion, longer disease duration, higher serum uric acid levels, bone erosion, and Achilles tendon involvement were closely associated with total DECT scores. DECT scoring system may be an invaluable tool for gout diagnosis owing to its high detection efficacy and a surrogate method to evaluate the amount of urate crystals and erosion of surrounding tissues.

## 1. Introduction

Gout is a common metabolic condition caused by the deposition of monosodium urate (MSU) crystals in joints and periarticular soft tissues. Overall, the first metatarsophalangeal (MTP^1st^) joint is the most commonly affected joint^[1]^ and the Achilles tendon is the most commonly affected tendon.^[2]^ Acute inflammatory flares result from the host response to MSU crystals and are probably triggered by crystal moving from the cartilage surface into the joint space.^[3]^ Increased deposition and a higher volume of urate crystals are associated with the development of joint destruction, renal dysfunction, cardiovascular and metabolic diseases, and increased mortality risk.^[4–6]^ Timely diagnosis and treatment initiation is crucial for better prognosis in patients with gout. The gold standard for the gout diagnosis is the identification of MSU crystals in joint fluid by arthrocentesis.^[7]^ However, paracentesis is seldom performed in clinical practice because of its invasiveness and risk of complications such as hemorrhage and infection.^[6]^ Recent studies have found that urate crystal deposition is present in some asymptomatic patients with hyperuricemia, suggesting that subclinical urate deposition occurs before the presentation of symptomatic disease.^[8,9]^

Clinical features of gout may be absent in some patients; therefore, imaging modalities can help identify affected joints and diagnose gout in such scenarios. Dual-energy computed tomography (DECT) has gained increasing importance in the diagnosis of gout over the past decade.^[10]^ DECT is a noninvasive alternative modality for detecting MSU crystals with high sensitivity, specificity, and diagnostic accuracy, especially in identifying subclinical tophi.^[10,11]^ Based on these characteristics, DECT was recently included in the 2015 European league against rheumatism/American college of rheumatology (EULAR/ACR 2015) classification criteria for gout.^[12]^ Moreover, MSU crystal deposition can be assessed using automated DECT-based volume assessment software in patients with gout receiving urate-lowering treatments. However, this urate volume assessment method is time-consuming and cannot easily measure the volume of specific deposition regions because a detailed analysis of specific regions significantly increases the time required for data generation.^[13]^

Given the limitations of automated urate volume measurement, Bayat et al^[13]^ has developed a semi-quantitative DECT urate scoring system that allows the measurement of MSU crystal deposits at specific sites of the feet and ankles. In this semi-quantitative scoring system, each scan was divided into 4 regions, and each region was scored as 0 to 3 in conformity with the maximum amount of urate deposition evaluated visually on color-coded 3-dimensional (3D) images of DECT. They found that DECT urate scores were highly correlated with urate volumes and this technique was less time consuming.^[13]^ In the majority of patients with gout, there is a foot and ankle involvement during the course of their disease^[14]^; therefore, this DECT urate scoring system allows for the efficient capture of clinically important information with less time and cost spent on scanning and scoring. While DECT has been increasingly used in the detection and volume assessment of MSU crystals in patients with gout, only 2 studies have applied this scoring system to evaluate urate deposition in these patients.^[15]^

In current study, we analyzed the total and 4 regions DECT scores of ankle/foot in different stages gout, to investigate the following hypothesis that this semi-quantitative DECT urate scoring system can evaluate MSU crystal deposition in the feet and ankles of patients with gout during different stages.

## 2. Methods

### 2.1. Study patients

**Figure 1.**
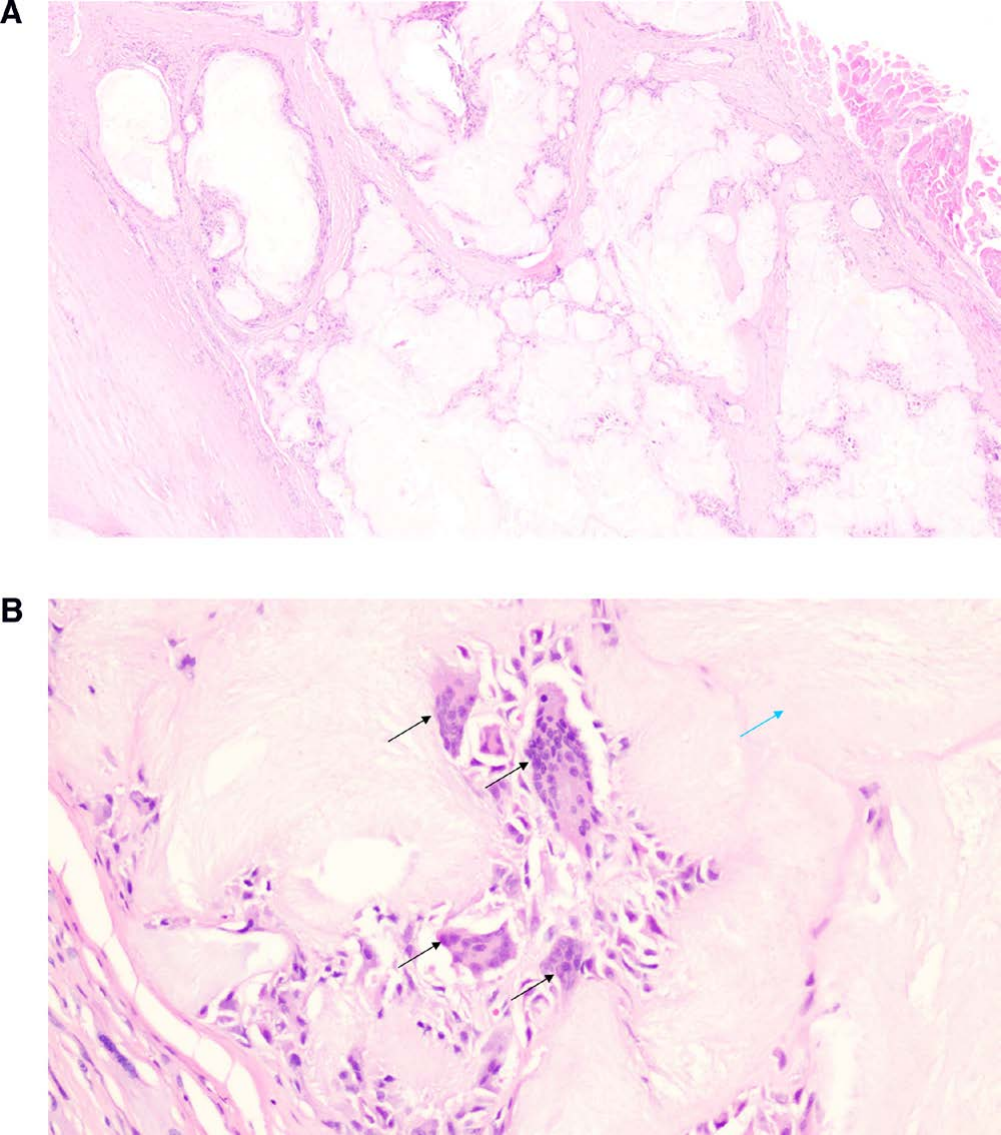
(a): H&E (2×) and (b): H&E (20×). Lesion is composed of multiple nodule foreign body granulomas. There are amorphous and acellular eosinophilic substances (urate crystals, as shown by the blue arrow) arranged in parallel or radially in the center of the granuloma, and multinuclear giant cells (as shown by the black arrows), fibrous tissues, and lymphocytes around.

In total, 287 patients with gout were recruited between November 2018 and November 2021. The inclusion criteria were as follows: patients aged 18 to 80 years; patients who underwent DECT examination of their feet and ankles; and patients diagnosed with gout based on the 2015 EULAR/ACR criteria^[12]^ and partly confirmed by arthroscopic debridement of the tophus (Fig. 1a–1b). The exclusion criteria were foot/ankle trauma and/or surgical procedures. This study was approved by the ethics committee of our hospital. Written informed consent was obtained from all participants. Patients were divided into 3 groups based on the duration of gout: early stage (≤1 year), middle stage (1–3 years), and late stage (>3 years).^[16]^ Clinical and serological data including age, sex, serum uric acid (SUA) and serum creatinine (SC) levels at the time of DECT (the interval between the test and performance of DECT within 7 days), bone erosion, and Achilles tendon involvement were recorded.

### 2.2. DECT examination

DECT examinations were performed using a 3-generation dual-source CT scanner (Somatom Definition Flash; Siemens Healthcare) equipped with 2 X-ray tubes and 2 corresponding detectors. All patients underwent a DECT scan of their feet and ankles in 1 acquisition. The scan parameters were set as follows: tube A, 140 kV/ 65mA; tube B, 80 kV/234 mA; pitch, 0.7; collimation, 128 × 0.6 mm; gantry rotation time, 1.0 second; reconstructed slice thickness/increment, 0.75 mm/0.5 mm; matrix, 512 × 512. Automated attenuation-based tube current modulation was used in all DECT examinations, with an average radiation doses of 10.3 mGy (0.03 mSv) for the feet and ankles. DECT post-processing was performed using a dedicated automated volume assessment software (Gout, Syngo CT Workplace, Siemens Medical Systems). The volume-rendered 3D images in which MSU crystal deposition was color-coded as green were reconstructed with a bone tissue convolution kernel (B70f). Multiplanar colored images, including the transverse, sagittal, and coronal planes, were reconstructed using a soft tissue kernel (B30f). These images allowed a visual display of MSU crystal deposition.

### 2.3. Image analysis

**Figure 2.**
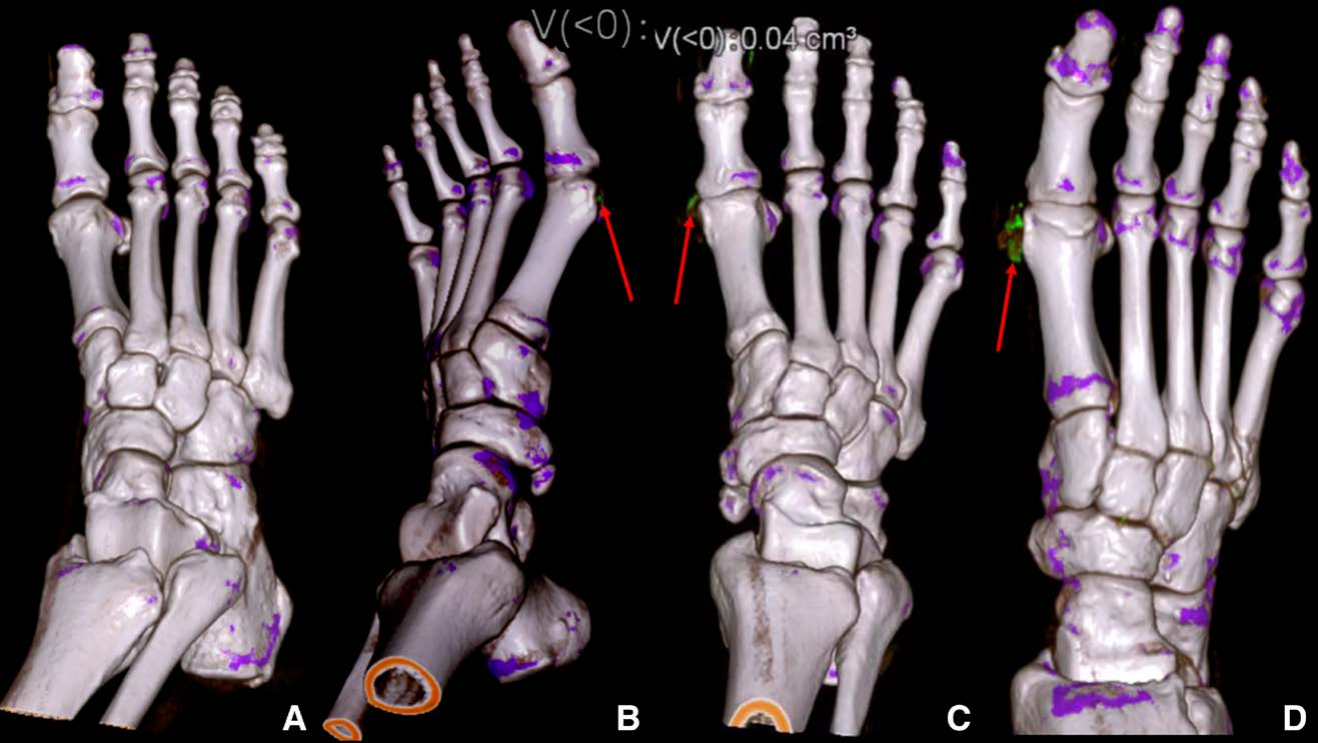
3D reconstruction DECT images of the first metatarsophalangeal joints with urate indicated as green (as shown by the red arrows). (a), score of 0; (b), score of 1; (c), score of 2; (d), score of 3. DECT = dual-energy computed tomography.

Two musculoskeletal radiologists, each with more than 9 years of experience in clinical magnetic resonance imaging (MRI), were blinded to the patients’ information and independently reviewed the acquired images. A third musculoskeletal radiologist with more than 24 years of experience reviewed the images to achieve a consensus when the former 2 observers had differences in reviewing images. The patient was diagnosed with gout on the condition that only 1 positive MSU crystal was discovered in a single joint. In the DECT urate scoring system, the feet and ankles are divided into 4 regions: the first metatarsophalangeal (MTP^1st^) joints, 2nd to 5th MTP (MTP^2nd–5th^) and 1st to 5th interphalangeal joints (ITP^1st–5th^), ankle/midfoot, and tendon/soft tissues. Each region is scored as follows: 0, no deposit; 1, punctiform deposit; 2, single deposit; and 3, more than 1 deposit (Fig. 2a–2d). The maximum score is 12 per ankle/foot.

### 2.4. Statistical analysis

Statistical analyses were performed using SPSS Statistics (version 22.0, SPSS Inc., Chicago, IL) and MedCalc (version 12.3, MedCalc Software, Mariakerke, Belgium). The inter-rater agreement between the 2 readers for DECT urate score evaluation was assessed using Cohen’s kappa statistic (*κ*). Categorical variables were presented as counts with percentages and compared using *χ*^2^ statistics or Fisher’s exact test, as appropriate. Continuous variables were described as mean and standard deviation for normally distributed data and as medians with quartiles for non-normally distributed data. Data distribution was examined using the Kolmogorov-Smirnov test. Continuous variables with normal distributions were compared using Student’s *t*-test or 1-way analysis of variance. Quantitative parameters with skewed distributions were compared using the non-parametric Mann-Whitney *U*-test. Pearson’s correlation coefficients were used to assess the correlations between the DECT urate scores and clinical and serological variables. The Wilcoxon Mann-Whitney test was used to analyze the associations between subgroups and study variables (total DECT urate scores, age, sex, bone erosion, disease duration, SUA level on DECT, SC level on DECT, and Achilles tendon involvement). Receiver operating characteristic analysis was conducted to assess the diagnostic performance of DECT urate scores for differentiating gout at different stages. Two-tailed *P* values of < .05 were considered statistically significant.

## 3. Results

### 3.1. Interobserver agreement in imaging analysis

Evaluation of the DECT urate scores showed excellent interobserver reproducibility. Interobserver agreement showed a *κ* value of 0.934 (95 % confidence interval [CI], 0.897–0.971) for the total DECT urate score. In addition, the agreement analysis showed that the *κ* values were 0.919 (95 % CI, 0.872–0.966) in the MTP^1st^ joint, 0.917 (95 % CI, 0.866–0.968) in the MTP^2nd to 5th^ and ITP^1st to 5th^ joints, 0.925 (95 % CI, 0.884 to 0.966) in the ankle/midfoot, and 0.945 (95 % CI, 0.914 to 0.976) in the tendon/soft tissue, respectively.

### 3.2. Clinical and laboratorial characteristics

The clinical and laboratorial characteristics were summarized in Table 1. Among the recruited patients, 93 (mean age, 38.38 ± 12.08 years) had early-stage gout, 70 (mean age, 41.81 ± 13.89 years) had middle-stage gout, and 124 (mean age, 44.90 ± 12.66 years) had late-stage gout. There was a significant difference in the mean age between patients in the early stage and patients in the late stage (*P *< .001). There were no significant differences in sex (male/female: 91/2 vs 65/5 vs 121/3, all *P *> .05) and mean SUA levels (523.40 vs 532.09 vs 518.05 *μ*mol/L, all *P *> .05) in different disease durations. The mean SC levels in the early stage was significantly lower than that in the late stage (84.46 vs 102.63 *μ*mol/L, *P* = .021). Bone erosion was significantly less in the early stage than in the late stage (5.4 % vs 15.3 %, *P* = .037). Achilles tendon involvements in the early and middle stages were significantly lower than that in the late stage (2.2 % vs 14.5 %, *P* = .004; 2.9 % vs 14.5 %, *P* = .019; respectively).

**Table 1:** Comparison of clinical and serological data of gout in different stages.

Variables	Early Stage (N = 93)	Middle Stage (N = 70)	Late Stage (N = 124)	*P* a	*P* b	*P* c
Age (yr)	38.38 ± 12.08	41.81 ± 13.89	44.90 ± 12.66	.091	.000	.107
Sex (male/female)	91/2	65/5	121/3	.120	.896	.112
Serum Uric Acid (*μ*mol/L)	523.40 ± 128.93	532.09 ± 150.61	518.05 ± 127.52	.682	.800	.506
Serum Creatinine (*μ*mol/L)	84.46 ± 17.11	90.24 ± 23.13	102.63 ± 88.22	.475	.021	.168
Bone Erosion	5(5.4%)	8(11.4%)	19(15.3%)	.194	.037	.511
Achilles Tendon Involvement	2(2.2%)	2(2.9%)	18(14.5%)	.779	.004	.019

a Early Stage vs Middle Stage.

b Early Stage vs Late Stage.

c Middle Stage vs Late Stage.

### 3.3. Comparison of DECT urate scores in different stages

Comparison of DECT urate scores of gout in different stages were presented in Table 2. There were significant differences in total and ankle/midfoot DECT urate scores between gouts at the 3 different stages (1.634 vs 2.500 vs 4.016; 0.183 vs 0.557 vs 0.992; all *P *< .05). The DECT urate score of the MTP^1st^ joint in the early stage was significantly lower than that in the late stage (0.409 vs 0.726, *P* = .030). The DECT urate scores of the MTP^2nd to 5th^ and ITP^1st to 5th^ joints in the early and middle stages were significantly lower than those in the late stage (0.215 vs 0.597, *P* = .001; 0.343 vs 0.597, *P* = .047, respectively). The DECT urate scores of tendon/soft tissue in the early and middle stages were significantly lower than those in the late stage (0.850 vs 1.702, *P *< .001; 1.071 vs 1.702, *P* = .001, respectively). No significant differences were observed between the positive presence of MSU crystals at the different stages (78.5 % vs 81.4 % vs 95.8 %, all *P *> .05).

**Table 2:** Comparison of DECT urate scores of gout in different stages.

Variables	Early Stage (N = 93)	Middle Stage (N = 70)	Late Stage (N = 124)	*P* a	*P* b	*P* c
Total	1.634 ± 1.465	2.500 ± 2.442	4.016 ± 3.100	.030	.000	.000
MTP^1st^ Joint	0.409 ± 0.887	0.529 ± 1.073	0.726 ± 1.164	.475	.030	.214
MTP^2nd–5th^ and ITP^1st–5th^ Joints	0.215 ± 0.568	0.343 ± 0.778	0.597 ± 1.043	.343	.001	.047
Ankles/Midfeet	0.183 ± 0.465	0.557 ± 0.987	0.992 ± 1.199	.015	.000	.003
Tendon/Soft Tissue	0.850 ± 1.113	1.071 ± 1.243	1.702 ± 1.294	.253	.000	.001
DECT (+)	73 (78.5%)	57 (81.4%)	118 (95.8%)	.877	.341	.479

a Early Stage vs Middle Stage.

b Early Stage vs Late Stage.

c Middle Stage vs Late Stage.

DECT = dual-energy computed tomography, ITP = interphalangeal, MTP** = **metatarsophalangeal.

### 3.4. Diagnostic efficacy of DECT urate scores for differentiating gout in different stages

**Figure 3.**
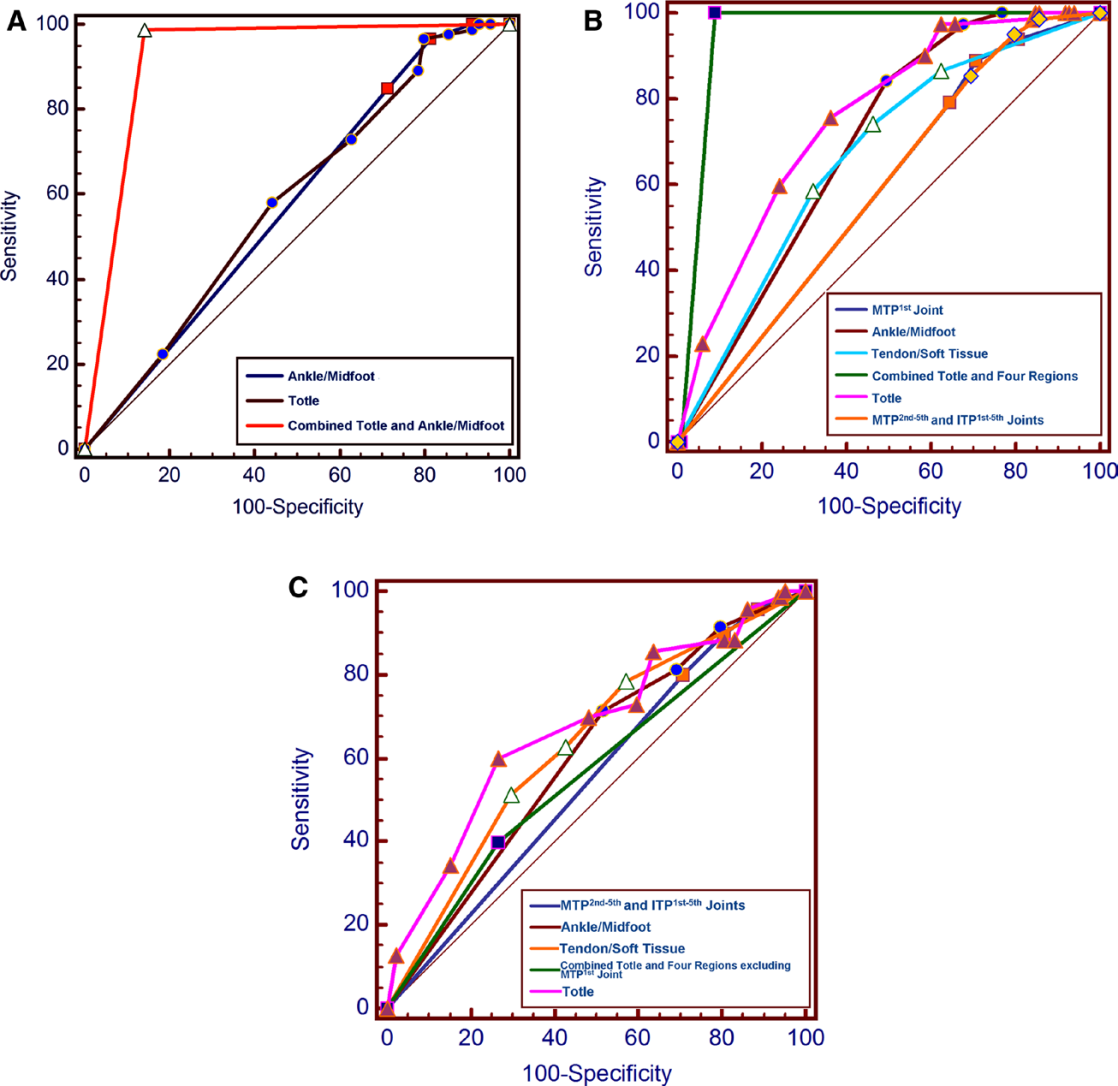
(a): ROC analysis of DECT urate scores for differentiating gout in the early stage from gout in the middle stage. (b): ROC analysis of DECT urate scores for differentiating gout in the early stage from gout in the late stage. (c): ROC analysis of DECT urate scores for differentiating gout in the middle stage from gout in the late stage. DECT = dual-energy computed tomography, ROC = receiver operating characteristics.

Table 3 and Figure 3a–3c show the receiver operating characteristics analyses for DECT urate scores in differentiating gout at different stages. When the total DECT urate score was ≤ 4, the sensitivity and positive predictive value (PPV) for distinguishing gout in the early and middle stages were 96.8% and 82.4%, respectively. When the ankle/midfoot DECT urate score was ≤ 1, the sensitivity and PPV for distinguishing gout in the early and middle stages were 97.8 % and 81.3 %, respectively. When total and ankle/midfoot DECT urate scores were combined, the area under the curve (AUC) was up to 0.923 for differentiating gout in the early stage from gout in the middle stage. When differentiating early-stage gout from middle-stage gout, the sensitivity, specificity, PPV, and negative predictive value (NPV) of combined total and ankle/midfoot DECT urate scores were 98.9 %, 85.7 %, 90.2 %, and 98.4 %, respectively.

**Table 3:** ROC analyses of DECT urate scores in differentiating gout in different stages.

Variables	AUC	Optimal cutoff	Sensitivity	Specificity	PPV	NPV
Total^a^	0.585	≤4	96.8%	20.0%	61.6%	82.4%
Ankles/Midfeet^a^	0.578	≤1	97.8%	18.6%	61.2%	81.3%
Combined Total and Ankles/Midfeet^a^	0.923	--	98.9%	85.7%	90.2%	98.4%
Total^b^	0.756	≤2	75.6%	63.6%	63.3%	75.9%
MTP^1st^ Joint^b^	0.586	≤1	89.0%	29.3%	51.0%	76.3%
MTP^2nd–5th^ and ITP^1st–5th^ Joints^b^	0.588	≤0	85.4%	30.3%	50.4%	71.4%
Ankles/Midfeet^b^	0.696	≤0	84.2%	50.5%	58.5%	79.4%
Tendon/Soft Tissue^b^	0.667	≤1	74.4%	53.5%	57.0%	71.6%
Combined Total and Four Regions^b^	0.949	--	100%	90.9%	90.1%	100%
Total^c^	0.673	≤2	60.0%	73.4%	56.0%	76.5%
MTP^2nd–5th^ and ITP^1st–5th^ Joints^c^	0.551	<0	0	100%	--	63.9%
Ankles/Midfeet^c^	0.604	<0	0	100%	--	63.9%
Tendon/Soft Tissue^c^	0.635	<0	0	100%	--	63.9%
Combined Total and Four Regions excluding MTP^1st^ Joint^c^	0.567	--	0	100%	--	63.9%

Four Regions include MTP1st Joint, MTP2nd–5th and ITP1st-5th Joints, Ankles/Midfeet, and Tendon/Soft Tissue.

a Early Stage vs Middle Stage.

b Early Stage vs Late Stage.

c Middle Stage vs Late Stage.

AUC = area under the curve, DECT = dual-energy computed tomography, ITP = interphalangeal, MTP = metatarsophalangeal, NPV = negative predictive value, PPV = positive predictive value, ROC = receiver operating characteristics.

When differentiating early-stage gout from late-stage gout, the AUC of total and 4 regions’ DECT urate score alone showed ordinary to moderate diagnostic efficacy of 0.586 to 0.756. However, when the total and the 4 regions’ DECT urate scores were combined, the AUC was up to 0.949 for differentiating early-stage gout from late-stage gout. When differentiating early-stage gout from late-stage gout, the sensitivity, specificity, PPV, and NPV of the combined total and the 4 regions’ DECT urate scores were 100 %, 90.9 %, 90.1 %, and 100 %, respectively.

When differentiating middle-stage gout from late-stage gout, the AUC of total, MTP^2nd to 5th^ and ITP^1st to 5th^ joints, ankle/midfoot, tendon/soft tissue DECT urate scores alone, and combined these 4 DECT urate scores showed the inferior diagnostic efficacy of 0.551 to 0.673. However, when the MTP^2nd to 5th^ and ITP^1st to 5th^ joints, ankle/midfoot, and tendon/soft tissue DECT urate scores were less than 0, the specificity of these 3 regions’ DECT urate scores alone and combined total and the 3 regions’ DECT urate scores were up to 100 % for differentiating middle-stage gout from late-stage gout.

### 3.5. Associations between total DECT urate score s and clinical characteristics

**Figure 4.**
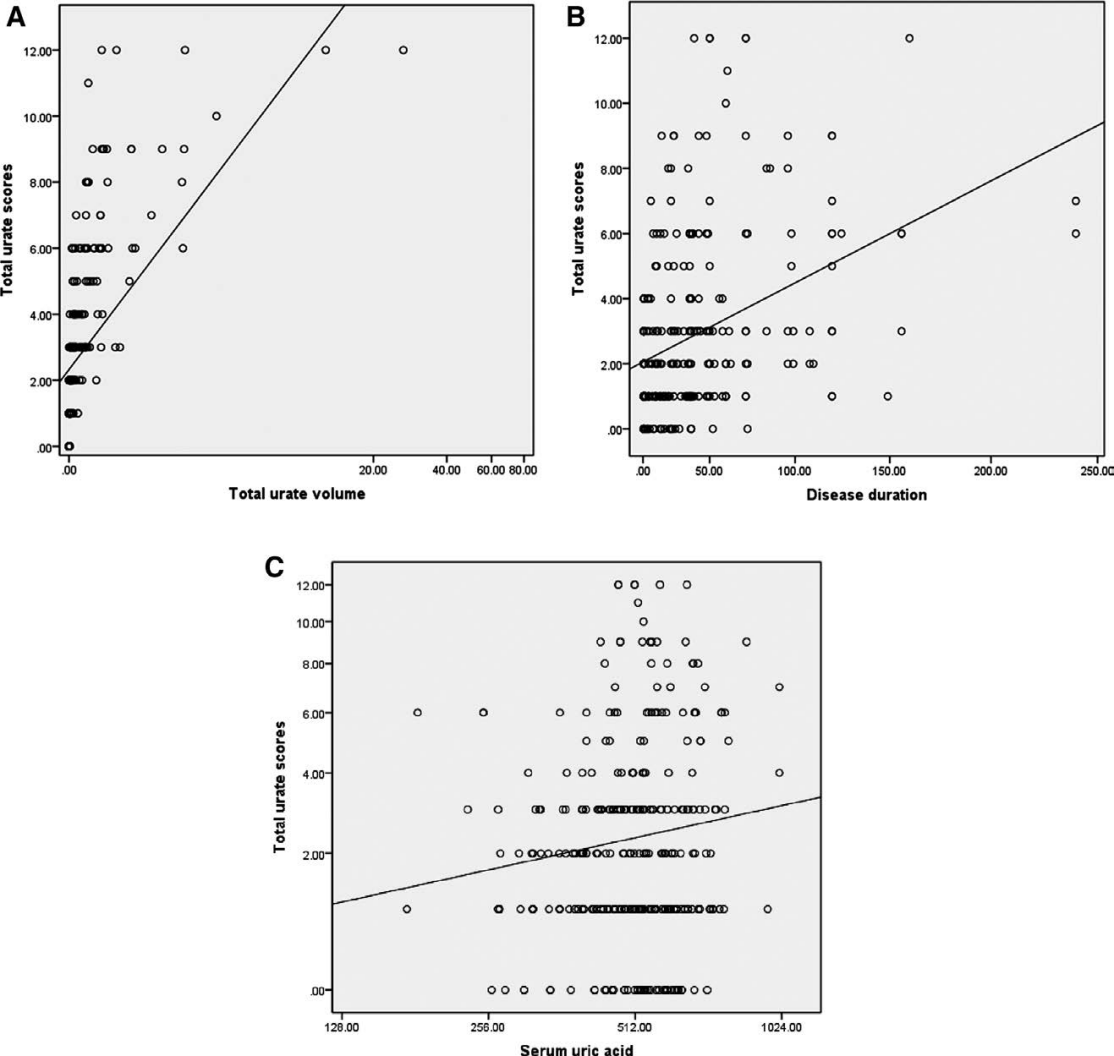
(a): Strongly positive correlation between the total DECT urate scores and volumes of urate crystal deposition. (b): Moderately positive correlation between the total DECT urate scores and disease duration. (c): Weakly positive correlation between the total DECT urate scores and SUA levels on DECT. DECT = dual-energy computed tomography, SUA = serum uric acid.

There was a highly positive correlation between the total DECT urate score and the volume of urate crystal deposition (*R* = 0.873, *P *< .001) (Fig. 4a). The total DECT urate scores were moderately positively correlated with disease duration (*R* = 0.393, *P* < .001) (Fig. 4b) and weakly positively correlated with SUA levels (*R* = 0.145, *P* = .008) (Fig. 4c).

Disease duration, mean SUA level on DECT, bone erosion, and Achilles tendon involvement significantly affected the total DECT urate score (all *P* < .01). However, age, sex, and SC levels on DECT did not significantly affect the total DECT urate scores (all *P* > .05). The detailed results are shown in Table 4.

**Table 4:** Associations between total DECT urate scores and clinical characteristics.

Variables	No.	DECT urate scores	*Z/t* a value	*P* value
Age (yr)			-1.199	.230
<42	157	2.65 ± 2.44		
≥42	130	3.17 ± 2.99		
Sex			-1.465	.143
male	277	2.91 ± 2.72		
female	10	1.90 ± 2.28		
SUA (*μ*mol/L) at DECT			-3.008	.003
≤410	57	2.03 ± 1.67		
>410	230	3.12 ± 2.90		
SC (*μ*mol/L) at DECT			-0.437	.662
≤110	237	2.85 ± 2.63		
>110	50	2.98 ± 3.12		
Bone Erosion			-5.969	.000
Yes	31	6.65 ± 3.86		
No	256	2.42 ± 2.14		
Achilles Tendon Involvement			-5.234	.000
Yes	22	7.09 ± 4.07		
No	264	2.53 ± 2.25		
Disease Durations (yr)			-6.267	.000
≤3	163	2.01 ± 1.99		
>3	124	4.02 ± 3.10		

a The *t* value and *Z* value were obtained by Student’s *t*-test or Mann-Whitney *U*-test, respectively, according to the results of the test for normal distributions.

DECT = dual-energy computed tomography, SUA = serum uric acid, SC = serum creatinine.

## 4. Discussion

This present study revealed that the level of SC and occurrence rate of bone erosion due to gout in the late stage were significantly higher than those in the early stage. This study also showed that the occurrence of Achilles tendon involvement in late-stage gout was significantly higher than that in early- and middle-stage gout. As the disease duration increased, an increasing number of MSU crystal deposits were present in the intra-articular and periarticular regions of the ankle/midfoot joints. The decline in renal function and development of joint destruction are generally due to long-term deposition of MSU crystals.^[5,6]^ Patients with positive DECT results have a higher frequency of renal insufficiency.^[17]^ Furthermore, higher volumes of MSU deposits detected by DECT had a high mortality risk and were associated with the development of cardiovascular and metabolic diseases.^[4]^ In addition, joint erosion is positively correlated with MSU crystal volumes.^[18]^ Therefore, prompt diagnosis and timely treatment of gout in the early stages are crucial for achieving a better prognosis. DECT is a highly accurate diagnostic tool for gout with high sensitivity and specificity.^[2,10,11]^ According to the 2015 EULAR/ACR criteria, positive DECT results for urate deposition can provide a 4-point score for diagnosing gout.^[12]^ In this present study, gout with positive DECT results for in the early, middle and late stages were 78.5 %, 81.4 %, and 95.8 %, respectively. Therefore, DECT may be a valuable tool for diagnosing gout at different stages.

DECT is an attractive diagnostic tool for gout owing to its advantages including automated urate volume measurement, 3-dimensional reconstruction features, and low radiation exposure.^[19]^ Color-coded images of DECT in 3 dimensions have proven to be sensitive enough to identify green MSU deposits in intra-articular and periarticular structures.^[2]^ A semi-quantitative DECT urate scoring system developed by Bayat et al^[13]^ allowed the visual evaluation of MSU crystal deposits on color-coded 3-dimensional images of DECT with less time consumption compared to automated urate volume measurement. They found that the DECT urate scores were strongly correlated with urate volumes.^[13]^ Similarly, the results obtained from our study demonstrated that the total DECT urate score was strongly positively correlated with the MSU deposit volumes. This present study demonstrated that the total DECT urate score was moderately associated with disease duration. The results of this study revealed that the total and 4 regions’ DECT urate scores became significantly higher as the disease duration was prolonged due to longer-term MSU crystal deposition. When combined with the total and ankle/midfoot or 4 regions’ DECT urate scores, the DECT urate scoring system achieved excellent diagnostic performance in differentiating gout in the early stage from gout in the middle and late stages (AUC, 0.923 and 0.949, respectively), with high sensitivity, specificity, PPV, and NPV (all were larger than 85%). When 4 regions excluding the MTP^1st^ joint alone or combined total and these 3 regions’ DECT urate scores, the optimal specificity (all were 100%) can be obtained in differentiating gout in the middle stage from that in the late stage. This DECT urate scoring system is easy to implement because green MSU crystals are visually easy to identify on color-coded 3-dimensional images of DECT. Based on these advantages, the DECT urate scoring system should be widely applyed in clinical practice.

Patients with higher volumes of MSU crystal deposits are likely to develop joint erosion.^[20]^ As our results showed, some studies demonstrated that the total DECT urate score was highly positively correlated with urate deposit volumes.^[13]^ Some studies have applied this urate scoring system and observed that a higher total urate score is closely associated with longer disease duration and bone erosion.^[15,19]^ The results of this present study are consistent with these findings. Furthermore, this present study demonstrated that longer disease duration and bone erosion were strongly associated with higher total DECT urate scores. In addition, our results showed that Achilles tendon involvement strongly affected the total DECT urate score. Dalbeth et al^[2]^ found that Achilles tendon was the most frequently involved tendon/ligament, which indirectly validats our results. When SUA concentrations were above the saturation levels (410 *μ*mol/L), MSU crystals formed at physiological temperature and pH.^[21]^ Increased urate concentrations were recognized as promoters of MSU crystals growth^[22]^ and were associated with higher MSU crystal depositions on DECT.^[18]^ This study demonstrated that patients with higher SUA levels on DECT had higher total DECT urate scores. The DECT urate score may be a surrogate biomarker to evaluate the amount of MSU crystal deposits and erosion of surrounding tissues.

However, this present study has some limitations. First, owing to the retrospective design of this study, there were selection biases in recruiting subjects. Second, a small number of gouts were confirmed using arthrocentesis because of the invasiveness of the procedure. Therefore, we used the 2015 EULAR/ACR criteria as a reference standard instead of the invasive method. Third, due to the nature of the cross-sectional study, we could not follow the progression of the disease and observe the clinical efficacy of urate-lowering treatment. Further longitudinal studies are required to better understand the characteristics of the disease. Finally, the DECT urate scoring system was not compared with other imaging diagnostic modalities. In the future, perspective studies with large sample sizes should compare the diagnostic efficacy of various imaging diagnostic modalities.

## 5. Conclusions

DECT may be a highly valuable tool for gout diagnosis at different stages owing to its high detection efficacy for MSU crystals. Longer disease duration, higher SUA levels on DECT, bone erosion, and Achilles tendon involvement were closely associated with higher total DECT urate scores. The DECT urate scoring system may be a surrogate method to evaluate the amount of MSU crystal deposits and erosion of surrounding tissues.

## Acknowledgements

The authors express sincerest gratitude to Longgang District Health Science and Technology Planning Project of Shenzhen Municipality (Award Number: LGKCYLWS2019000500) for grant support. This work was supported by the Special Talent Development Project of Shenlong Meritocrat of Shenzhen Longgang District.

## Author contributions

**Conceptualization:** Wanling Ma.

**Data curation:** Huanhuan Zhong, Minghua Wang, Heng Zhang, Zhitian Huang, Yuhui Zhu.

**Formal analysis:** Heng Zhang, Yiwen Liang, Yuhui Zhu.

**Funding acquisition:** Zhitian Huang, Wanling Ma.

**Investigation:** Nanai Xie.

**Methodology:** Guannan Zou.

**Project administration:** Nanai Xie, Wanling Ma.

**Resources:** Minghua Wang, Zhitian Huang, Baochang Zou, Guannan Zou.

**Supervision:** Wanling Ma.

**Validation:** Baochang Zou, Yiwen Liang.

**Visualization:** Baochang Zou, Guannan Zou, Yiwen Liang, Yuhui Zhu.

**Writing – original draft:** Huanhuan Zhong.

**Writing – review & editing:** Minghua Wang, Heng Zhang, Wanling Ma.
